# Oral Capsaicin as Treatment for Unexplained Chronic Cough and Airway Symptoms

**DOI:** 10.1016/j.chpulm.2024.100049

**Published:** 2024-03-20

**Authors:** Ewa Ternesten-Hasséus, Ewa-Lena Johansson, Eva Millqvist

**Affiliations:** a Department of Respiratory Medicine and Allergology, Institute of Medicine, The Sahlgrenska Academy, University of Gothenburg, Gothenburg, Sweden; b Department of Respiratory Medicine, Allergology and Palliative Medicine, Sahlgrenska University Hospital, Region Västra Götaland, Gothenburg, Sweden; c Department of Physiotherapy and Occupational Therapy, Sahlgrenska University Hospital, Region Västra Götaland, Gothenburg, Sweden

**Keywords:** capsaicin, chronic refractory cough, cough hypersensitivity, desensitization, refractory cough, tachyphylaxis, unexplained chronic cough

## Abstract

**Background:**

Unexplained chronic cough (UCC) is characterized by persistent coughing without evident medical explanation.

**Research Question:**

Does daily oral administration of natural capsaicin (chili) improve refractory coughing in patients with UCC?

**Study Design and Methods:**

Forty-six patients (mean age, 60.5 years) with UCC participated in this crossover, randomized double-anonymized study. For 4 weeks, the participants took capsules containing pure capsaicin orally, and after a washout of 2 weeks, took placebo capsules for 4 weeks. A capsaicin inhalation cough test was used to assess the capsaicin inhalation concentration required to reach two coughs and the capsaicin inhalation concentration required to reach five coughs. The number of coughs in a 24-h period and the number of coughs per hour were recorded on four occasions using the Leicester Cough Monitor. Participants completed questionnaires with items on cough, cough-related symptoms, and quality of life.

**Results:**

The mean values for capsaicin inhalation concentration required to reach two coughs and the capsaicin inhalation concentration required to reach five coughs increased after the capsaicin treatment period as compared with the first visit (baseline; *P* < .05 and *P* < .03, respectively), although they did not differ from the placebo recordings. Neither the capsaicin nor the placebo treatment significantly reduced the cough frequency, nor did they differ between the two treatment periods. When dividing the participants into low-cougher (≤ 400 coughs within 24 h) and high-cougher (> 400 coughs within 24 h) groups, the low coughers experienced a significantly better outcome from capsaicin, but not from placebo. The visual analog scale symptom scores improved after capsaicin treatment compared with baseline and placebo treatment in terms of the frequencies of coughing (*P < .*001), rhinitis (*P < .*03), and throat irritation (*P < .*01). The Leicester Cough Questionnaire scores improved after capsaicin treatment compared with baseline and compared with the placebo treatment group for all the domains (*P < .*01 for the total score).

**Interpretation:**

In this study, capsaicin powder taken orally was found to be clinically effective and well tolerated by patients with UCC. The results suggest a future treatment for UCC.

**Trial Registry:**

European Union Drug Regulating Authorities Clinical Trials Database; No.: EudraCT 2016-004463-39; URL: https://www.clinicaltrialsregister.eu

ClinicalTrials.gov; No.: NCT04125563; URL: www.clinicaltrials.gov

**Take-home Points**

**Study Question:** Does daily oral administration of natural capsaicin (chili) improve refractory coughing in patients with unexplained chronic cough?

**Results:** In this study, capsaicin powder taken orally was found to be clinically effective and well tolerated by patients with unexplained chronic cough.

**Interpretation:** The results suggest a future treatment for capsaicin in unexplained chronic cough.

Daily cough is regarded as chronic when it persists for more than 8 weeks.^1^ Such a cough, without evident findings and with repeated treatment failures, often is termed *unexplained* or *refractory* or is referred to as unexplained chronic cough (UCC).^1^ Inhaled capsaicin provokes cough and is, at least in healthy people and in patients with asthma, followed by a short-lasting desensitization in concordance with the capsaicin tachyphylaxis described in other studies.^2^, ^3^, ^4^ A recent study showed amelioration of chronic refractory cough when combining increasing doses of inhaled capsaicin and behavioral therapy.^5^ Others have shown good effects on UCC resulting from behavioral therapy.^6^, ^7^, ^8^ Anecdotally, some patients in our clinic stated that their ongoing, unexplained coughing disappeared when they returned temporarily to their home countries and consumed a spicy diet. In a pilot study, cough symptoms were improved by daily intake of capsaicin powder in capsules.^9^ Thus, we aimed to assess, in a more rigorous way, whether oral intake of capsicum oleoresin capsaicinoids (capsaicin; C_18_H_27_NO_3_) derived from chili extract could desensitize the cough reflex and relieve unexplained coughing through tachyphylaxis.

## Study Design and Methods

Fifty-five patients with UCC^1^ were randomized at the Asthma and Allergy Clinic, Sahlgrenska University Hospital, Gothenburg, Sweden. The inclusion and exclusion criteria are listed in Table 1. A statistical analysis plan was set up that described the intended clinical trial analyses. The treatment period order in this crossover trial was randomized using block randomization with a nonspecified block size, overseen by independent statisticians. An accredited manufacturer prepared the labeling with etiquette marked and coded in accordance with the randomization scheme. The key that identified the patients and to which group they belonged was held by a third party at Sahlgrenska University Hospital, and it was not revealed to the researchers until the study was closed.

**TABLE 1:** Inclusion and Exclusion Criteria for Assigned Participants

**Inclusion criteria**
Outpatients, men and women
Age 18-75 y
Presently does not smoke, with a maximum smoking history of ≤ 10 pack-y
Diagnosis of unexplained chronic cough made by a trained specialized physician
Exceeding the cutoff limit for the Swedish version of the Hull Airway Reflux Questionnaire (a total score of ≥ 13 points)
At screening visit, reporting daily, troublesome coughing and an easily evoked cough reflex for at least 2 mo
At screening visit, positive results for capsaicin inhalation cough test
At screening, FEV_1_ > 70% predicted
**Exclusion criteria**
Known or suspected chili allergy
Known or suspected allergy to the colorant tartrazine (FD&C yellow)
Any kind of diabetes
Treatment the preceding month with any kind of chili medication or food supplement containing capsaicin or having a diet including chili in treatment purpose
Treatment the preceding month with angiotensin-converting enzyme (ACE) inhibitors or any kind of codeine or opioids
Pregnancy, breastfeeding, or planned pregnancy
Suspected poor capability to follow instructions of the study
Airway infection the last 4 wk before study start
Any significant disease, disorder, or abnormal findings that, in the opinion of the investigator, may influence the patient’s ability to participate in the study
Smoking during the last 10 y, for > 10 pack-y, or both
Known alcohol or drug misuse, or both
Study start date adjusted to seasonal allergy or another possible allergen exposure

Capsules, taken orally, that contained standardized doses of capsicum oleoresin, corresponding to 0.4 mg of pure capsaicin per capsule, were compared with matched capsules with placebo. The capsaicin doses chosen were based on the daily intake levels in countries that have a spicy diet^10^^,^^11^ and on the experience gained from a previous pilot study.^9^ Thus, 4 weeks of active treatment were compared with 4 weeks of placebo treatment with a washout of 2 weeks in between. For the first 2 weeks of each 4-week period, the patients took one capsule bid, and this was followed by two capsules bid for 2 weeks.

**Figure 1:**
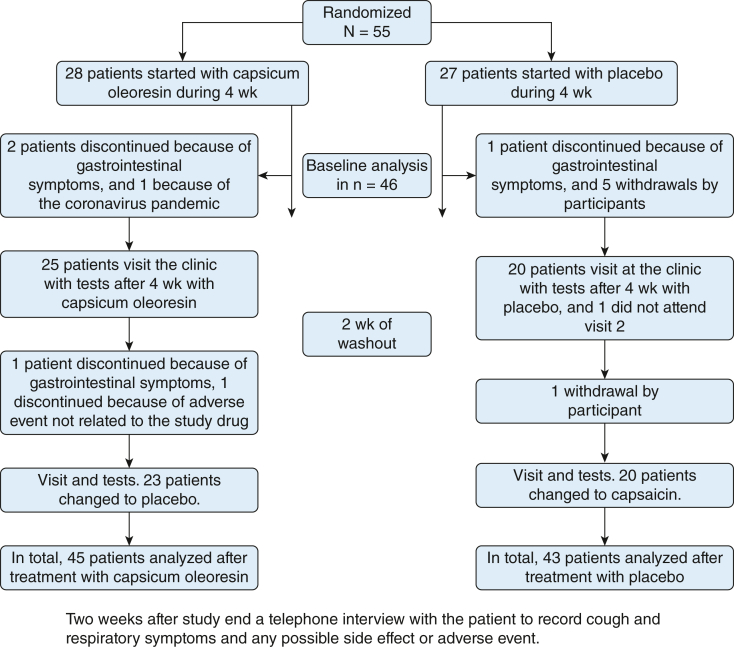
Flowchart for the study.

The patients visited the clinic on four occasions for about 1.5 h (Fig 1). Written informed consent was obtained from all participants after they were provided with information about the study in both verbal and written formats. All data were documented in an electronic case report form. An independent monitor from the Western Sweden health care region (Gothia Forum) supervised the study. The Swedish Ethical Review Authority and the Swedish Medical Products Agency approved the study. The Swedish Ethical Review Authority also approved the protocol changes required because of the COVID-19 pandemic (Identifier: 5.1-2019-9058 and 2021-04320).

### Capsaicin Capsule Formulation

The formulation was developed by Research Institutes of Sweden. The agent was prepared from capsicum oleoresin 8.4% (content of total capsaicinoids expressed as capsaicin, 8.0%-8.8%). Each soft capsule contained one dose corresponding to 0.4 mg of capsaicin. All test methods used were according to the European Pharmacopoeia for Capsicum Oleoresin, refined and standardized.^12^ The supplier of raw material for the capsicum oleoresin product was Ransom Naturals Ltd. The herbal substance is produced in line with the European Union Good Agricultural and Collection Practices (European Medicines Evaluation Agency and Committee on Herbal Medicinal Products Identifier: 246816/2005).^13^ The specification for the herbal substance is in accordance with the European Pharmacopeia monograph for capsicum.^12^ The manufacture, quality control, and filling of soft capsules with capsicum oleoresin were carried out by Catalent Pharma Solutions Inc.. The placebo product was a soft capsule, identical in form and color to the investigational medical product. The filling material was 400 mg glycerol monocaprylocaprate and a colorant (FD&C yellow [tartrazine]) was used. Packing bottles of capsules with the investigational medical product was carried out by Apotek Produktion & Laboratorier AB. Labeling was carried out by Tamro AB.

### Primary Outcomes

Changes in the values of the inhaled capsaicin inhalation concentration required to reach two coughs (C2) and capsaicin inhalation concentration required to reach five coughs (C5) from baseline to the end of week 4 for both the active period and the placebo period, and the effect comparison for these periods, were noted. At each visit, in accordance with the flowchart (Fig 1), cough sensitivity was assessed by a standardized capsaicin inhalation cough test using the threshold method,^14^ following the guidelines of the European Respiratory Society.^15^ Lung function was measured with spirometry.

### Secondary Outcomes

The secondary outcomes included changes from baseline to the end of week 4 regarding the Leicester Cough Monitor (LCM)^16^ variables for both the active and the placebo treatment periods and an effect comparison for these periods. The LCM assessed the cough frequency for a 24-h period on four separate occasions, analyzed as the number of total coughs and the number of coughs per hour.^16^ Cough detection largely was automated using specifically designed software. The analyses were performed at King’s College, London, England.

The secondary outcomes also included changes from baseline and comparisons between the capsaicin and placebo treatment periods for the results of the patient symptom estimation for the week before baseline, as well as the last week of the capsaicin and placebo treatments. Six symptoms (cough, rhinitis, heavy breathing, throat irritation, stomach problems, and stress urinary incontinence) were scored on a visual analog scale (VAS) of 0 to 100, with 0 indicating no symptoms and 100 indicating intense symptoms.^17^

As an additional secondary outcome, at each visit, the patients provided responses to the Swedish version of the Leicester Cough Questionnaire (LCQ-S)^18^^,^^19^ and the Swedish version of the Hull Airway Reflux Questionnaire,^20^^,^^21^ and comparisons with the baseline and between the outcomes of the respective periods with capsaicin and placebo were made.

### Safety Variables

For the safety parameters, the participants completed a diary of symptoms deemed relevant to the study. All the safety analyses were descriptive according to treatment period and were documented in the electronic case report form. A safety report was completed after the study ended and was submitted to the Swedish Ethical Review Authority and to the Swedish Medical Products Agency.

### Statistical Analyses

As described in the statistical analysis plan, to reach the power of 0.80 with mean difference in logC2 between the active placebo treatment of 0.590 and SD for the difference of 0.885, with a two-tailed Fisher nonparametric permutation test, α = 0.025, 24 patients were needed to be included in the study. To reach the power of 0.80 with mean difference between in logC5 the active-placebo treatment of 0.425 and SD for mean difference of 0.911, with two-tailed Fisher nonparametric permutation test, α = 0.025, 47 patients were needed to be included in the study. To adjust for dropouts, 50 patients would be included. Because of problems associated with the COVID-19 pandemic, we randomized 55 patients to account for any dropouts.

All randomized patients with follow-up measurements for at least one period were included in the intention-to-treat population. All enrolled patients who received at least one dose of the randomized capsaicin or placebo treatment were included in the safety population.

The main analyses were performed on the intention-to-treat population, and all the main analyses also were tested for period effects. For the tests regarding the effects of capsaicin vs placebo, all prespecified outcomes of interest were evaluated using the independent *t* test and the dependent *t* test to understand the within effect from baseline to follow-up for each group. To handle the potential sequencing and period effects, a linear mixed model was estimated for each end point. Based on this model, the overall effect is presented together with the corresponding *P* value.^22^ For subgroup analyses, nonparametric tests were used. All data were analyzed using SAS software version 9.1 or later (SAS Institute, Inc.) or IBM SPSS version 22 software (IMB SPSS Inc.), and the results were considered statistically significant if the *P* value was < .05.

## Results

Of the 55 randomized patients, nine patients did not complete at least one full treatment period, so 46 patients were included in the intention-to-treat analyses in accordance with the power calculation in the statistical analysis plan. Overall, 28 patients started with capsaicin treatment and 27 started with placebo treatment. During the first weeks of the study, three participants who started with capsaicin discontinued and six participants dropped out during the placebo period (Fig 1). The participants’ characteristics at baseline are presented in Table 2. Because of carryover effects, we used the prerandomization (very first visit) values as baseline, while we also made comparisons with period-dependent baseline values.

**TABLE 2:** Participants’ Characteristics at Baseline

Variable	Overall
No. of participants	46
**Sex**	
Female	38 (82.6)
Male	8 (17.4)
Age, y	60.5 ± 8.9
BMI, kg/m^2^	26.1 ± 4.8
Duration of cough, y	18.9 ± 11.4
**Smoking history**	
Never smoked	38 (82.6)
Previously smoked	8 (17.4)
Pack-y of smoking	4.5 ± 3.3
**Cough induced by**	
Chemical substances	42 (91)
Cold air	35 (76)
**Comorbidity**	
Asthma (assessed by PI)	5 (10.9)
Rhinitis VAS score > 5	39 (85)
Throat irritation VAS score > 5	41 (89)
High BP (assessed by PI)	7 (15.2)
Gastritis or Reflux (assessed by PI)	5 (10.9)
Stress urinary incontinence VAS score > 5	20 (44)
Heart disease (assessed by PI)	3 (6.5)
Migraine	1 (2.2)
**Symptoms in the past 1 wk (VAS 0-100)**	
Cough	63.4 ± 27.9
Rhinitis	41.4 ± 30.8
Throat irritation	46.5 ± 30.0
Gastro-intestinal symptoms	15.4 ± 24.7
Stress urinary incontinence	20.6 ± 31.1
**Drugs at baseline**	
Short-acting β2 agonist	14 (30.4)
Inhaled corticosteroids	7 (15.2)
Inhaled corticosteroids plus long-acting β2 agonist	11 (23.9)
Nasal steroids	4 (8.7)
Leukotriene receptor antagonist	4 (8.7)
Antihistamines	4 (8.7)
Proton pump inhibitor	16 (34.8)
**Spirometry**	
FEV_1_ % predicted	101.7 ± 15.5

Data are presented as No. (%) or mean ± SD unless otherwise indicated. PI = principal investigator; VAS = visual analog scale.

The spirometry values were within normal limits at the starting point and did not differ significantly from baseline during the study (data not shown). To address the great variation in the patients for 24-h coughs at baseline (range, 22-2657 coughs) we divided the cohort into: low coughers, with ≤ 400 coughs within a 24-h period (n = 23) and high coughers, with > 400 coughs within a 24-h period (n = 20).

### Primary Analyses of Efficacy

The values for the logarithmic capsaicin cough thresholds at baseline and after the capsaicin and placebo administrations are shown in Table 3. The C2 and C5 values were significantly higher after the period of capsaicin treatment as compared with the cough thresholds at the first visit (baseline), but did not differ compared with values obtained after placebo administration. At baseline, the low coughers did not differ significantly from the high coughers for C2 or C5 values. The C2 and C5 values after capsaicin treatment were higher for the low coughers than for the high coughers (*P* < .009 and P < .006, respectively).

**TABLE 3:** Effects of Treatment (ITT Population) for Capsaicin Cough Sensitivity and Cough Counting

End Point	No.	Mean ± SD	*P* Value^a^	Overall Effect	*P* Value^b^
**Primary**					
**C2 logarithmic values**					
Baseline	46	0.6 ± 1.0			
Capsaicin	45	1.0 ± 1.01	.049	0.89	.87
Placebo	43	0.9 ± 1.1	.251	0.91	
**C5 logarithmic values**					
Baseline	46	1.16 ± 0.9			
Capsaicin	45	1.7 ± 1.3	.026	1.57	.50
Placebo	43	1.7 ± 1.4	.029	1.64	
**Secondary**					
**LCM cough/h entire group**					
Baseline	43	22.2 ± 20.7			
Capsaicin	45	17.9 ± 18.5	.31	19.44	.89
Placebo	43	17.4 ± 15.9	.24	19.25	
**LCM cough/24 h entire group**					
Baseline	43	531 ± 519.4.			
Capsaicin	45	413 ± 422.3	.235	458.92	.981
Placebo	43	412 ± 368.5	.213	458.17	
**LCM cough/h in low coughers**^c^					
Baseline	23	8.4 ± 4.7			
Capsaicin	22	6.1 ± 4.3	0.03^d^		.05^e^
Placebo	23	8.6 ± 6.6	1.0^d^		
**LCM cough/h in high coughers**^f^					
Baseline	20	37.7 ± 21			
Capsaicin	20	30.5 ± 20.9	0.004^d^		.306^e^
Placebo	18	28.1 ± 18.4	0.003^d^		

C2 = capsaicin inhalation concentration required to reach two coughs; C5 = capsaicin inhalation concentration required to reach five coughs; ITT = intention-to-treat; LCM = Leicester Cough Monitor.

a Within effect from baseline to follow-up.

b Based on a linear mixed model adjusted for period and sequencing.

c Wilcoxon signed-rank test used because of small groups. Low coughers defined as ≤ 400 coughs/24 h.

d *P* value vs baseline.

e Outcomes from the capsaicin treatment period vs placebo period.

f Wilcoxon signed-rank test used because of small groups. High coughers defined as > 400 coughs/24 h.

For the low coughers, the C2 and C5 values increased (*P* < .03 and *P* < .01, respectively) after capsaicin treatment compared with baseline and the C5 value increased after placebo administration (*P* < .04), although the results after capsaicin treatment did not differ compared with those after placebo administration. Among the high coughers, no significant differences were noted.

### Secondary Analyses of Efficacy

Regarding the overall differences in LCM values for the entire group, neither capsaicin nor placebo administration significantly reduced the mean number of total coughs in a 24-h period or the mean number of coughs per hour, and the cough counts did not differ between the two treatment periods (Table 3). Compared with the high coughers, the low coughers reported significantly fewer coughs for a 24-h period and fewer coughs per hour at baseline, both after the capsaicin treatment and after placebo period (*P* < .001 for all).

Analyzing the high coughers and the low coughers separately (Table 3) showed that, compared with baseline, the low coughers reported significantly fewer coughs in a 24-h period and fewer coughs per hour after capsaicin treatment (*P* < .03 for both), although not after the placebo period. The numbers of coughs per hour differed significantly between the period after capsaicin treatment and after placebo administration (*P* < .05). For the high coughers, both capsaicin and placebo significantly reduced the number of coughs in a 24-h period and the number of coughs per hour, although no statistically significant difference between the two periods was noted.

We compared the overall differences in the participants’ estimations of the previous week’s symptoms with the VAS scores during the 10 weeks of the study (Table 4). The overall effect for the VAS scores improved significantly after the capsaicin treatment period as compared with baseline and after placebo administration. Comparing the high coughers with the low coughers showed significantly lower VAS cough scores for the low coughers at baseline, after capsaicin treatment, and after placebo administration (*P* < .01 for all). For the low coughers, the VAS cough scores improved after the capsaicin period (*P* < .01), but not after placebo administration, and the scores did not differ in between these administrations. Among the high coughers, no differences were found with respect to VAS cough scores after capsaicin treatment compared with baseline and after placebo administration.

**TABLE 4:** VAS Score (0-100) the Week Before Baseline and the Last Week of Each Treatment Period

VAS Score (Secondary End Points)	No.	Mean ± SD	*P* Value^a^	Mean Score Change	Overall Effect of Capsaicin	*P* Value^b^ Capsaicin vs Placebo
**Coughing**						
Baseline	46	63.4 ± 27.9				
After capsaicin	44	47.0 ± 32.8	.012	16.4	50.2	.001
After placebo	43	53.1 ± 28.3	.087	10.3	50.7	
**Rhinitis**						
Baseline	46	41.4 ± 30.8				
After capsaicin	44	32.7 ± 30.4	.181	8.7	32.0	.029
After placebo	43	31.0 ± 28.5	.100	10.0	37.5	
**Heavy breathing**						
Baseline	46	32.0 ± 34.2				
After capsaicin	44	23.5 ± 29.2	.205	8.5	26.3	.590
After placebo	43	22.7 ± 28.4	.169	9.3	25.2	
**Throat irritation**						
Baseline	46	48.4 ± 30.5				
After capsaicin	43	30.0 ± 30.0	.005	18.4	32.6	.010
After placebo	43	34.3 ± 30.4	.031	14.1	39.1	
**Gastrointestinal symptoms**						
Baseline	46	15.6 ± 24.6				
After capsaicin	44	24.5 ± 26.5	.102	–8.9	21.8	.179
After placebo	43	14.3 ± 21.8	.769	1.3	18.6	
**Stress urinary incontinence**						
Baseline	46	22.0 ± 33.1				
After capsaicin	44	16.8 ± 28.3	.424	5.2	19.4	.007
After placebo	43	23.4 ± 34.6	.851	–1.4	23.0	

VAS = visual analog scale.

a Within effect from baseline to follow-up.

b Based on a linear mixed model adjusted for period and sequencing.

Analysis of the overall effects on LCQ-S revealed that the total score and all three Leicester Cough Questionnaire domains improved significantly after capsaicin treatment, and they differed compared with baseline and after placebo administration (Table 5). After the capsaicin treatment period, the mean improvement in total LCQ-S was 2.2 points. The mean total LCQ-S increased by 1.5 points after placebo administration (Table 5).

**TABLE 5:** LCQ-S the Week Before Baseline and Last Week of Each Treatment Period

LCQ-S Score (Exploratory End Points)	No.	Mean ± SD	*P* Value^a^	Mean Score Change	Overall Effect	*P* Value^b^
Total LCQ-S score						
Baseline	46	11.7 ± 3.6				
After capsaicin	44	13.9 ± 3.8	.008	2.2	13.3	.004
After placebo	43	13.2 ± 3.8	.053	1.5	12.6	
**Physical domain LCQ-S score**						
Baseline	46	4.0 ± 1.2				
After capsaicin	44	4.5 ± 1.3	.029	0.5	4.37	.033
After placebo	43	4.3 ± 1.2	.172	0.3	4.2	
**Psychological domain LCQ-S score**						
Baseline	46	4.1 ± 1.4				
After capsaicin	44	4.9 ± 1.4	.008	0.8	4.7	.019
After placebo	43	4.7 ± 1.3	.032	0.6	4.4	
**Social domain LCQ-S score**						
Baseline	46	3.7 ± 1.4				
After capsaicin	44	4.5 ± 1.6	.011	0.8	4.3	.001
After placebo	43	4.2 ± 1.5	.079	0.5	3.9	

A low LCQ-S score indicates an impaired quality of life. LCQ-S = Swedish version of the Leicester Cough Questionnaire.

a Within effect from baseline to follow-up.

b Based on a linear mixed model adjusted for period and sequencing.

Comparing the high coughers with the low coughers revealed a significantly higher (better) total LCQ-S score for the low coughers at baseline, after capsaicin exposure, and after placebo administration (*P* < .01 for all). For the low coughers, the total LCQ-S cough scores improved after the capsaicin treatment period (*P* < .01) and after placebo administration (*P* < .02), but did not differ in between these periods. The high coughers improved only after capsaicin exposure (*P* < .05).

We compared the Swedish version of the Hull Airway Reflux Questionnaire scores after 4 weeks of capsaicin treatment or placebo administration and with the corresponding scores at baseline. The mean Swedish version of the Hull Airway Reflux Questionnaire score decreased from 37 to 31 (*P < .*05) after capsaicin treatment, whereas no differences in outcome were noted between the two periods with capsaicin treatment or placebo administration, and the scores did not differ between high coughers and low coughers.

### Safety Assessment

The participants recorded each day in a diary (using the VAS) all the symptoms related to cough and other concerns. The weekly results are presented in Table 4. One severe adverse event occurred when a patient experienced intense muscular chest pain after a heavy lift; she was observed in hospital for 24 h, but no serious cause (no heart affection) for the pain was found. Overall, 56% of the patients during the capsaicin treatment period experienced gastrointestinal symptoms that could be related to the treatment, whereas 33% experienced gastrointestinal symptoms during the placebo period. Most common were gastric acid symptoms or reflux and epigastric pain. In most cases, the gastrointestinal symptoms arose in relationship to ingestion of the capsules, lasted only a short time, and were of mild or moderate character and were transient.

## Discussion

The main results can be summarized as follows. First, we showed that the primary effect parameter, cough sensitivity to inhaled capsaicin, does not differ significantly from the effect of the placebo. The group of participants characterized as low coughers (≤ 400 coughs within a 24-h period) tended to show decreased cough sensitivity to inhaled capsaicin after oral capsaicin treatment, but not after placebo. Second, the secondary effect parameter of cough counts assessed with LCM after the capsaicin treatment did not differ in comparison with the number of coughs recorded after placebo treatment regarding the entire group. When separating the patients into high coughers and low coughers, compared with baseline, the low coughers reported significantly fewer coughs in a 24-h period and fewer coughs per hour after capsaicin treatment, but not after the placebo period. Moreover, the number of coughs per hour differed significantly after capsaicin treatment compared with after placebo administration. This indicates that the definition of UCC may include various phenotypes, and a crucial determinant may be the number of coughs in a 24-h period.

Third, the self-assessed parameters (questionnaires), which were also part of the secondary outcomes, revealed amelioration of cough and other symptoms after capsaicin treatment as compared with baseline and after placebo administration. The LCQ-S scores and VAS scores for cough, rhinitis, throat irritation, and stress urinary incontinence improved significantly after capsaicin treatment, and they differed from the values obtained at baseline and after placebo treatment.

The improvements in VAS scores for rhinitis and throat irritation may mirror mechanisms linked to cough symptoms associated with increased sensitivity in the entire airway system. Regarding urinary stress incontinence, we cannot deduce whether this incontinence is originally the result of the UCC or a symptom that is worsened by chronic coughing and that the improvement of this parameter is the result of less coughing. Because the mean VAS cough scores decreased 16 points, they did not reach the level deemed to indicate a clinically important change, as defined by Nguyen et al.^23^ However, we regard the significant cough VAS improvement seen in the current study as important, although the low number of patients may have influenced the results.

After the capsaicin treatment period, the mean improvement in total LCQ-S was 2.2 points, indicating a clinically important change that is in accordance with the result (improvement of ≥ 1.3 points) reported by Nguyen et al.^24^ Based on the meta-analysis conducted by Zheng et al,^25^ the LCQ and the VAS scores for cough in the present study are comparable with the results obtained from several other studies of cough medications. Other authors have disclosed significant improvements in LCQ of 2.5 to 5.32 points for various treatments.^5^^,^^26^, ^27^, ^28^

As observed in several cough studies, a clear placebo effect exists^29^, ^30^, ^31^ that may be dependent on central mechanisms, although this is not yet understood fully.^31^, ^32^, ^33^, ^34^ The strong placebo effects seen on the current study’s parameters indicate an influence on central mechanisms as shown in other cough studies.^31^^,^^35^^,^^36^

We hypothesize that oral capsaicin desensitizes not only the Transient Receptor Potential Vanilloid 1 (TRPV1) receptors, but also other TRPV receptors involved in the cough reflex,^2^ with consequent amelioration of coughing. The ability to get become accustomed to spicy food and the fact that topical capsaicin can ameliorate neuropathic pain^37^ suggest that an improvement of UCC may be possible through desensitizing or exhausting the TRPV receptors’ cough signals, which is in concordance with the capsaicin tachyphylaxis demonstrated in several studies.^2^^,^^4^ The recent study conducted by Slovarp and Bozarth^38^ showed a combination of behavioral therapy and inhalation of increasing doses of capsaicin even after 3 weeks improves coughing and, especially, the urge to cough. This suggests that UCC cough treatment should be combined with behavioral therapy, such as that provided by a speech-language pathologist.^8^^,^^39^

Given the high variability observed for the coughing patterns of many patients, a 24-h cough measurement and an inhaled irritant cough test performed on separate occasions may not capture the daily cough symptomatology adequately. Positive, masked results based on the patients’ own estimations of their condition for 1 week may provide a more accurate picture from the clinical viewpoint.^40^

In the present study, although at the baseline visit only five patients were judged to have asthma by the principal investigator (E.M.), 21 of the patients were receiving some form of asthma medication. Although five patients were considered to have gastritis or gastric reflux, 16 of the patients were using proton pump inhibitors. Although whether these medications influenced the present results is not known, the patients did not experience satisfactory outcomes from the therapies, despite often being treated for years.

Limitations of this study include the overall low number of participants and the low number of male participants. Also a need exists for long-term follow-up regarding possible adverse events and tolerance to the study drug.

In summary, capsaicin powder taken orally was shown to ameliorate UCC and was well tolerated. The findings indicate a future treatment possibility for UCC. The question arises as to whether the capsaicin products that currently are available in health food and drug stores can provide relief for patients with UCC. The answer to this is that these products, to the best of our knowledge, are not certified or validated in the appropriate way, and the capsaicin concentrations, the cultivation, and the production processes are uncertain.

## Funding/Support

This study was supported by grants from Vinnova, Sweden’s innovation governmental foundation agency; the Swedish state under the agreement between the Government of Sweden and the County Councils; the ALF agreement (Agreement on Medical Education and Research). [Identifier, ALFGBG771164]; the Health & Medical Care Committee of the 10.13039/100007212Region Västra Götaland, Sweden; the 10.13039/501100003793Swedish Heart and Lung Foundation; the Swedish Cancer and Allergy Fund; and the Swedish Asthma and Allergy Association’s Research Foundation. The entire study was financed exclusively by research grants with no industrial involvement.

## Financial/Nonfinancial Disclosures

Professor Millqvist filed an international patent application (PCT application) for the use of capsaicin as a cough reducing product on January 3, 2014. There is a pending patentapplication in US. In Australia, Canada and EU a patent was issued in 2017. Professor Millqvist has lectured for several pharmaceutical companies and participated in an expert group on chronic cough in the MSD regimen. Professor Millqvist has no conflicts of interest regarding cough monitors. None declared (E. T. H. and E.-L. J).
